# Assessment of genetic diversity and population structure of oil palm (*Elaeis guineensis* Jacq.) field genebank: A step towards molecular-assisted germplasm conservation

**DOI:** 10.1371/journal.pone.0255418

**Published:** 2021-07-29

**Authors:** Siou Ting Gan, Chin Jit Teo, Shobana Manirasa, Wei Chee Wong, Choo Kien Wong

**Affiliations:** 1 Biotechnology Section, Advanced Agriecological Research Sdn. Bhd. (AAR), AAR-UNMC Biotechnology Research Centre, Semenyih, Selangor, Malaysia; 2 Plant Breeding Section, Advanced Agriecological Research Sdn. Bhd. (AAR), Paloh, Johor, Malaysia; Institute of Mediterranean Forest Ecosystems of Athens, GREECE

## Abstract

Oil palm (*Elaeis guineensis*) germplasm is exclusively maintained as *ex situ* living collections in the field for genetic conservation and evaluation. However, this is not for long term and the maintenance of field genebanks is expensive and challenging. Large area of land is required and the germplasms are exposed to extreme weather conditions and casualty from pests and diseases. By using 107 SSR markers, this study aimed to examine the genetic diversity and relatedness of 186 palms from a Nigerian-based oil palm germplasm and to identify core collection for conservation. On average, 8.67 alleles per SSR locus were scored with average effective number of alleles per population ranging from 1.96 to 3.34 and private alleles were detected in all populations. Mean expected heterozygosity was 0.576 ranging from 0.437 to 0.661 and the Wright’s fixation index calculated was -0.110. Overall moderate genetic differentiation among populations was detected (mean pairwise population *F*_*ST*_ = 0.120, gene flow *Nm* = 1.117 and Nei’s genetic distance = 0.466) and this was further confirmed by AMOVA analysis. UPGMA dendogram and Bayesian structure analysis concomitantly clustered the 12 populations into eight genetic groups. The best core collection assembled by Core Hunter ver. 3.2.1 consisted of 58 palms accounting for 31.2% of the original population, which was a smaller core set than using PowerCore 1.0. This core set attained perfect allelic coverage with good representation, high genetic distance between entries, and maintained genetic diversity and structure of the germplasm. This study reported the first molecular characterization and validation of core collections for oil palm field genebank. The established core collection via molecular approach, which captures maximum genetic diversity with minimum redundancy, would allow effective use of genetic resources for introgression and for sustainable oil palm germplasm conservation. The way forward to efficiently conserve the field genebanks into next generation without losing their diversity was further discussed.

## Introduction

Oil palm (*Elaeis guineensis* Jacq.) is the most productive oil crop, accounting for 40% of the global production of vegetable oils, and producing 3–8 times more oil per hectare as compared to other oil crops with planting on only 0.4% of global agricultural land [1]. Increased production of oil crops is essential for global food security to meet the demand of growing human population. The world demand for vegetable oil is estimated to reach 240 million tonnes by 2050 [2]. Oil palm with its high productivity and lowest production cost has the potential to meet this increased oil demand with minimum additional adverse effect to the environment.

Despite being originated from Africa, oil palm is mostly cultivated in Southeast Asia with large plantations established in Malaysia and Indonesia due to favourable tropical climates [3]. Commercial oil palm has narrow genetic base originating from a small number of ancestral palms, which is referred to as “breeding population of restricted origins” (BPROs) [4]. The mother palm, Deli *dura*, is descended from only four palms planted in Bogor Botanic Gardens in 1848 in Java, Indonesia [5]. Seeds from an ancestral “Djongo” palm in Congo were planted in Sumatra, Indonesia by AVROS (Algemeene Vereniging van Rubberplantera ter Oostkust van Sumatra) in 1923 and subsequently gave rise to AVROS *pisifera* that is widely used as father palm in Indonesia, Malaysia, Papua New Guinea (PNG) and Costa Rica [6]. In view of the narrow genetic base, the Malaysian Palm Oil Board (MPOB) has carried out extensive collection of oil palm genetic materials from its centre of origin in Africa and Latin America (for *Elaeis oleifera*) since 1970s [7]. The objective is to broaden the genetic base for further breeding to meet future demands. The germplasms are maintained as *ex situ* living collections in the field and Malaysia currently has the largest oil palm field genebank in the world [8].

Conservation of genetic resources is crucial because it provides a reservoir of genes for development of novel traits associated with yield enhancement, adaptability to climate change as well as pest and diseases resistance/tolerance in crop plantation. Hence plants genetic resources are important and useful for improvement of productivity and sustainability of the agricultural production systems [9]. *Ex situ* field genebanks provide an easy and ready access to the resources for utilization, characterization and evaluation. However, they require large land area and are expensive and labour intensive to maintain, especially for perennial crops *i*.*e*., oil palm. The living collections are also threatened by extreme weather conditions such as flood, drought as well as various pest and diseases in the field that may lead to potential loss of the invaluable genetic resources. In order to efficiently conserve and exploit the genetic resources, understanding of the genetic diversity and population structure of the oil palm germplasm is essential. Conservation of minimal number of accessions with maximal genetic diversity/ minimal genetic redundancy, namely the core collection, would reduce the maintenance cost of oil palm germplasm (land, labour, fertilizers, etc.) and allow for sustainable germplasm conservation. Genetic diversity of germplasm can be investigated using various techniques such as morphological assessment, biochemical markers (isozymes) and molecular markers. With rapid development of molecular technology, genetic evaluation of oil palm populations has been conducted using various markers available, for example, isozymes [10], restriction fragment length polymorphism (RFLP) [11, 12], amplified fragment length polymorphism (AFLP) [12], simple sequence repeats (SSR) [13–18], and single nucleotide polymorphism (SNP) [19]. The advancement of next-generation sequencing techniques and publication of oil palm genome sequence in 2013 [20] have further accelerated markers development, particularly high-density SNP markers, that are useful for genetic diversity studies of oil palm as well as quantitative trait loci (QTL) and genome-wide association studies [21, 22]. Sequence data could be mapped to the oil palm reference genome for SNP calling and identification of genome distribution of markers. Nevertheless, SSR markers, also known as microsatellites, have been widely applied in genetic diversity studies of oil palm due to its multi-allelic nature, high specificity, codominance, genome abundance and reliability.

In AAR, exotic germplasms were collected from different sources via collaboration and are currently being maintained as field genebanks. One of the collections are Nigerian-based germplasm which represents PORIM (Palm Oil Research Institute of Malaysia, now known as MPOB)’s selection for short stature and high yielding materials for productions of their PS1 DxP materials [23]. The PS1 planting materials are known for their slow height increment (20–45 cm/year), high fresh fruit bunch (FFB) yield (30–33 t/ha/year) and high oil to bunch (O/B) ratio (approximately 28%). These materials were disseminated to AAR in 1990s for evaluation and exploitation. The morphological and agronomical traits have shown the potential of this Nigerian germplasm for introgression such as high oil yield, short palm stature with slim petiole and short frond for high density planting, virescent fruit color for ease of harvesting and high IV value for palm oil quality improvement. The main objectives of this study were to characterize the genetic diversity and population structure of the Nigerian-based germplasm using molecular markers and to identify core set to provide clues to develop efficient conservation strategies to bring current germplasm to the next generation without losing their genetic diversity *i*.*e*., molecular-assisted germplasm conservation.

## Materials and methods

### Plant materials and DNA isolation

The Nigerian-based germplasm collection, which descended from the MPOB Nigerian population 12, was planted in 1995 at AAR oil palm breeding research station in Paloh Estate, Johor, Malaysia. A total of 186 progenies from 12 controlled-crosses were assayed in the present study (Table 1).

**Table 1:** Summary of the AAR’s Nigerian-based germplasm collection assayed in this study.

Cross no.	Origin	Mating Nature	No. of palms
**596**	0.150/501 x 0.150/500	Sibs-mating	20
**597**	0.150/1908 x 0.150/665	Sibs-mating	8
**598**	0.150/1968 x 0.150/1908	Sibs-mating	19
**599**	0.150/499 x 0.149/13131	Cross-mating	12
**600**	0.149/14674 x 0.150/2036	Cross-mating	20
**601**	0.150/2333 self	Selfing	8
**602**	0.150/484 self	Selfing	13
**603**	0.150/1477 x 0.150/1908	Sibs-mating	18
**604**	0.150/499 self	Selfing	5
**605**	0.150/2360 self	Selfing	12
**606**	0.150/2334 self	Selfing	31
**607**	0.150/2356 self	Selfing	20

The leaflets of the first fully-opened frond were harvested for genomic DNA isolation using the QIAGEN^®^ DNeasy Plant Mini kit according to manufacturer’s instruction (QIAGEN, Germany). The concentration and quality of the DNA samples were examined using a Nanodrop One^C^ Spectrophotometer (Thermo Fisher Scientific, USA) and agarose gel electrophoresis. The stock DNA samples were diluted to a working concentration of 10 ng/μl using sterile water.

### Microsatellite genotyping

A set of 107 *E*. *guineensis* microsatellite markers were used for genotyping in this study, comprising 103 markers developed at CIRAD (Centre de Coopération Internationale en Recherche Agronomique pour le Développement; French Agricultural Research Centre for International Development) [24, 25] and four at MPOB [26, 27] (S1 Table). The SSR markers were widely distributed throughout the 16 chromosomes of oil palm genome, indicating a good genome coverage for studying its genetic diversity and population structure (S1 Table). The forward SSR primers were fused with a M13 (-21) tail, 5’-TGT AAA ACG ACG GCC AGT-3’ (18 bp) at the 5’-end. The PCR amplification was performed in a 10 μl reaction mixture containing 20 ng DNA, 5 μl HotStarTaq^®^ *Plus* Master Mix (QIAGEN, Germany), 4 μM M13-tagged forward primer, 4 μM reverse primer, and 0.4 μM IRDye^®^-labelled M13 (-21) primer. PCR reactions were performed with an initial denaturation of 95°C for 5 minutes, followed by 35 cycles of denaturation at 94°C for 30 s, annealing at 52°C for 1 min., extension at 72°C for 1 min., with a final extension of 72°C for 10 min. The thermocycling was performed with Veriti^™^ Thermal Cycler (Applied Biosystems, USA). The PCR products were mixed with 7 μl of loading buffer (98% formamide (v/v), 0.1% bromophenol blue (w/v) and 0.01 M EDTA pH 8.0) and heat-denatured. The PCR products were then separated on 6% polyacrylamide gels using the infrared dye detection system NEN 4300 DNA Analyzer (Li-COR Biosciences, USA).

### Data analysis

Clearly visualised fragments detected using NEN4300 DNA Analyzer were examined and data were scored manually in a binary format (1 or 0). Software GenAlEx version 6.503 [28, 29] was used to analyse genetic diversity parameters, including allele frequencies, observed number of alleles (*A*), number of effective alleles (*A*_*e*_), number of private alleles (*PA*), Shannon’s Information Index (*I*), observed and expected heterozygosity (*H*_*o*_ and *H*_*e*_), and fixation index (*F*) for each locus and population.

Cluster analysis based on the unweighted pair-group with arithmetic averaging (UPGMA) was performed using the multivariate statistical package MVSP (Kovach Computing Services, Anglesey, Wales) to construct a dendogram. Principal Coordinate Analysis (PCoA) was carried out using GenAlEx version 6.503 to further analyse the genetic relationship among individual palms of all the populations. The genetic distance between populations according to [30] and pairwise population *F*_*ST*_ value were also computed. Analysis of Molecular Variance (AMOVA) was performed to calculate the molecular variance among and within the populations with the number of permutations = 999.

Population structure of the Nigerian-based germplasm was analysed using the Bayesian model-based clustering programme STRUCTURE version 2.3.4 [31]. The model was run with a burn-in period of 10,000 steps followed by 50,000 Monte Carlo Markov Chain (MCMC) replicates for *K* set from 1 to 12. The most likely number of populations (*K*) was determined via the *ad hoc* statistic Δ*K* [32].

### Construction and validation of core collections

PowerCore 1.0 [33] and Core Hunter version 3.2.1 [34] software were used for development of independent core collections using the genotypic data. Maximization strategy was first proposed by [35], which maximized the number of observed alleles in the dataset. The advanced maximization (M) strategy with modified heuristic search was implemented in PowerCore to select the most diverse palm accessions to represent the entire set of alleles in the collection [33]. Core Hunter uses local search algorithms to generate core subsets relying on one or more criteria, including genetic distance and genetic diversity measures [36]. Core Hunter version 3.2.1 was executed through R package (https://cran.r-project.org/package=corehunter). Core Hunter was applied to maximize allelic coverage index (CV) in order to retain all unique alleles in the original populations for current work.

The different core sets established were compared based on (i) proportions of alleles retained in the core collection; (ii) average genetic distance between each accession in the original collection and the nearest entry in the core set (A-NE); (iii) average distance between entries (E-E); (iv) minimum and average distance between each entry and the nearest neighbouring entry (E-NE), as proposed by [37]. A-NE indicates representativeness of the core sets and the value should be as small as possible. Whereas, E-NE and E-E indicate the dispersion of entry in the core sets with higher value achieved when diverse entry were sampled. Although Core Hunter utilizes Modified Roger’s genetic distance [38, 39] as default for core set sampling, Bray-Curtis genetic distance [40] was applied in current work for unbiased comparison of core sets obtained from both software. Additionally, genetic diversity parameters were calculated for both the core collections and compared against the genetic diversity of the entire germplasm using Student’s T-test. If the p-value was greater than 0.05, the difference between the core and entire population was considered to be non-significant. Allele frequency for all loci was also compared between both core collections and entire germplasm using Chi-square test (p<0.05). UPGMA dendograms were constructed for both core collections to examine the genetic relationship among individual core palms, with those reported for the germplasm populations.

## Results

### Polymorphism of SSR markers

The evaluated 107 SSRs detected a total of 928 alleles across the 186 Nigerian-based germplasm, with an average of 8.67 alleles per loci (Table 2). Both the mEgCIR0350 and mEgCIR2215 SSRs generated the lowest and highest number of alleles per SSR, 3 and 16, respectively. PIC values were observed to differ significantly, ranging from 0.155 (mEgCIR3300) to 0.710 (mEgCIR3362), with a mean value of 0.525. 65% of the markers had PIC values more than 0.5, suggesting that the evaluated markers are highly informative for genetic diversity evaluation [41].

**Table 2:** Summary of genetic diversity calculated for the 107 SSR markers used in genotyping the 186 palms.

	Allele No.	Expected Heterozygosity	Polymorphic Information Content (PIC)
**Mean**	8.67	0.576	0.525
**Max**	16	0.749	0.710
**Min**	3	0.172	0.155

### Genetic diversity of Nigerian-based populations

The genetic variability parameters of each Nigerian-based population are summarised in Table 3. The mean number of different alleles (*A*) and effective alleles (*A*_*e*_) within each population was 4.290 and 2.777, respectively. Population 606 with the highest samples size (*N* = 31) recorded the highest number of alleles (*A* = 6.365) whereas pop. 599 exhibited the highest number of effective alleles (*A*_*e*_ = 3.343). Population 604 with the lowest number of palms (*N* = 5) and a selfed-crossing nature revealed the lowest in number of different alleles (*A* = 2.654), effective alleles (*A*_*e*_ = 1.964), Shannon’s Information Index (*I* = 0.729) and expected heterozygosity (*H*_*e*_ = 0.437). Private alleles were detected in all 12 populations with the lowest and highest in pop. 598 (*PA* = 1) and 606 (*PA* = 33), respectively. Population 606 recorded the highest Shannon’s Information Index (*I* = 1.322). The heterozygosity in the Nigerian-based populations was generally high with an average observed (*H*_*o*_) and expected heterozygosity (*H*_*e*_) of 0.644 and 0.576, respectively. Except for pop. 605 (*F* = 0.161), *H*_*o*_ consistently exceeded *H*_*e*_ leading to negative fixation index (*F*) in the studied populations, which reflected excess of heterozygotes.

**Table 3:** Genetic diversity among the Nigerian-based populations.

Cross	Origin	N	A	A_e_	PA	I	H_o_	H_e_	F
**596**	0.150/501 x 0.150/500	20	5.533	3.179	9	1.299	0.662	0.645	-0.026
**597**	0.150/1908 x 0.150/665	8	3.374	2.567	7	0.977	0.619	0.547	-0.111**
**598**	0.150/1968 x 0.150/1908	19	3.991	2.726	1	1.039	0.689	0.572	-0.190***
**599**	0.150/499 x 0.149/13131	12	5.224	3.343	9	1.318	0.690	0.661	-0.030
**600**	0.149/14674 x 0.150/2036	20	5.336	3.137	10	1.234	0.655	0.619	-0.043
**601**	0.150/2333 self	8	2.766	2.379	2	0.858	0.694	0.514	-0.328***
**602**	0.150/484 self	13	4.785	2.891	11	1.153	0.658	0.588	-0.106***
**603**	0.150/1477 x 0.150/1908	18	3.561	2.690	5	1.006	0.734	0.578	-0.253***
**604**	0.150/499 self	5	2.654	1.964	4	0.729	0.510	0.437	-0.158***
**605**	0.150/2360 self	12	4.645	2.764	4	1.143	0.501	0.592	0.161***
**606**	0.150/2334 self	31	6.365	3.233	33	1.322	0.687	0.642	-0.071**
**607**	0.150/2356 self	20	3.243	2.454	9	0.906	0.633	0.522	-0.192***
		**Mean**	4.290	2.777	8.667	1.082	0.644	0.576	-0.110

*N* = Number of palms; *A* = Number of different alleles; *A*_*e*_ = Number of effective alleles; *PA* = Number of private alleles; *I* = Shannon’s Information Index; *H*_*o*_ = Observed heterozygosity; *H*_*e*_ = Expected heterozygosity; *F* = Fixation index.

**significant at the 1% level,

*** significant at the 0.1% level.

Overall values are averages for *A*, *A*_*e*_, *I*, *H*_*o*_, *H*_*e*_ and *F*; Total for PA.

### Genetic relatedness and structure

The genetic distance calculated among the Nigerian-based populations revealed close genetic relationship (mean = 0.466). The largest genetic distance was observed between pop. 601 and 597 (0.698) whereas the closest genetic relationship was between pop. 598 and 603 (0.242) (Table 4). Both analysis of molecular variance (AMOVA) and pairwise *F*_*ST*_ analysis were performed to investigate the genetic variations among the populations. High genetic differentiation was observed among pop. 604 with pop. 597, 598, 599, 600, 601, 603 and 607 (*F*_*ST*_ = 0.153–0.202) as well as among pop. 607 with pop. 597 and 601 (*F*_*ST*_ = 0.166 and 0.174, respectively) (Table 4). Population 597 was also genetically distant from pop. 601 (*F*_*ST*_ = 0.177) while the remaining populations have moderate genetic differentiation (*F*_*ST*_ = 0.066–0.150). The average genetic differentiation among populations was 0.120 and the overall gene flow value (*Nm*) was estimated at 1.117, indicating certain level of alleles migration among populations. AMOVA analysis further disclosed that 71% of molecular variance observed was due to the genetic difference within population, whereas 29% genetic variation was partitioned among populations.

**Table 4:** Pairwise population matrix of Nei’s genetic distance (below diagonal) and *F*_*ST*_ value (above diagonal) among the Nigerian-based populations.

**Population**	**596**	**597**	**598**	**599**	**600**	**601**	**602**	**603**	**604**	**605**	**606**	**607**
**596**	0.000	0.111	0.081	0.078	0.084	0.097	0.073	0.084	0.128	0.079	0.066	0.112
**597**	0.462	0.000	0.139	0.115	0.132	0.177	0.136	0.143	0.195	0.144	0.126	0.166
**598**	0.321	0.522	0.000	0.094	0.119	0.096	0.117	0.072	0.182	0.136	0.107	0.146
**599**	0.388	0.505	0.392	0.000	0.077	0.123	0.090	0.111	0.158	0.109	0.084	0.119
**600**	0.379	0.553	0.497	0.349	0.000	0.121	0.076	0.125	0.153	0.102	0.095	0.136
**601**	0.344	0.698	0.292	0.493	0.454	0.000	0.115	0.092	0.185	0.142	0.117	0.174
**602**	0.293	0.536	0.465	0.396	0.293	0.397	0.000	0.116	0.137	0.092	0.083	0.099
**603**	0.342	0.560	0.242	0.500	0.539	0.281	0.454	0.000	0.173	0.126	0.108	0.150
**604**	0.430	0.680	0.637	0.603	0.545	0.577	0.425	0.596	0.000	0.123	0.125	0.202
**605**	0.348	0.591	0.595	0.527	0.439	0.537	0.359	0.534	0.362	0.000	0.075	0.142
**606**	0.312	0.560	0.470	0.432	0.451	0.465	0.357	0.487	0.412	0.318	0.000	0.117
**607**	0.424	0.599	0.531	0.478	0.533	0.602	0.333	0.555	0.669	0.544	0.458	0.000

**Fig 1:**
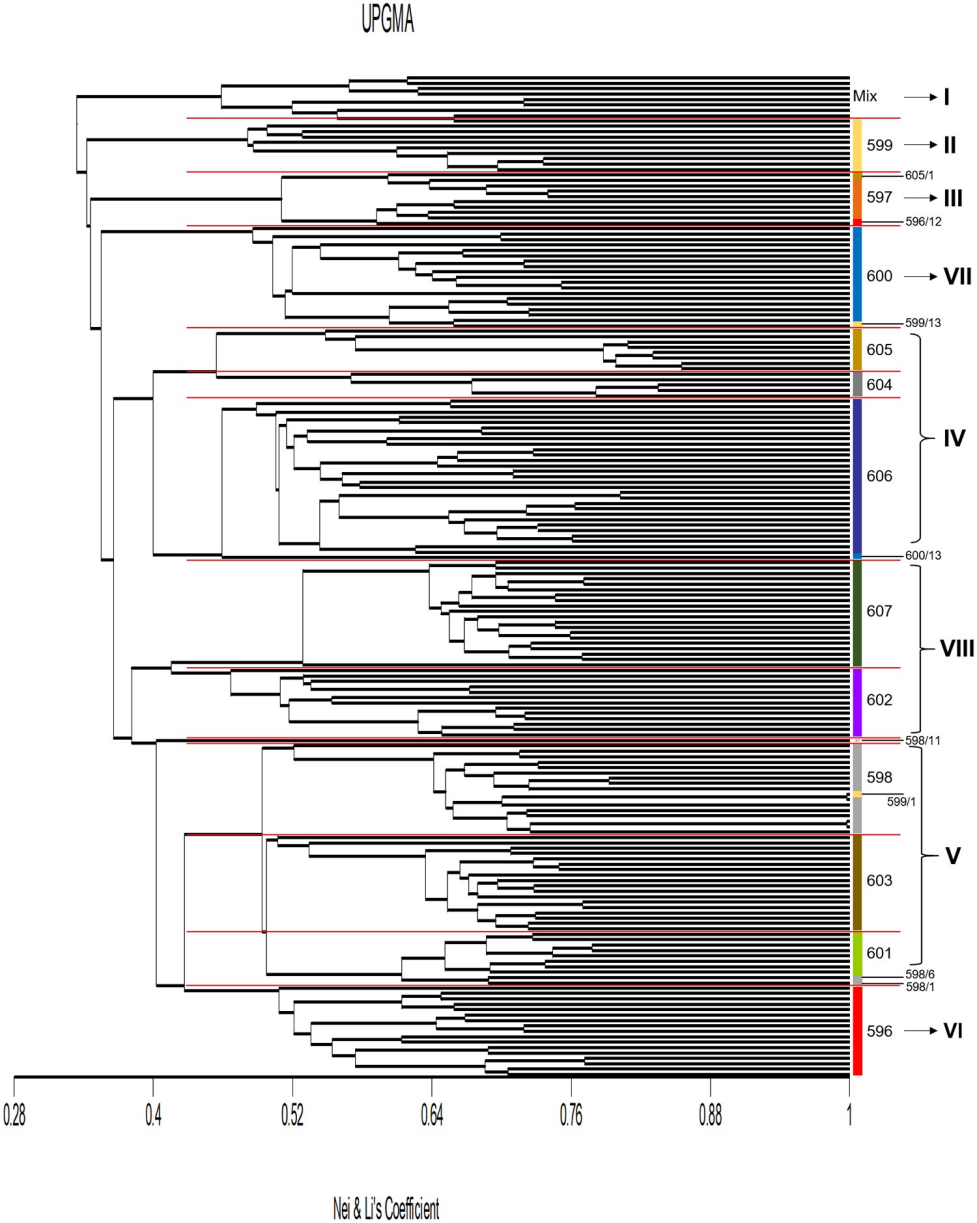
UPGMA dendogram based on Nei and Li’s coefficient.

**Fig 2:**
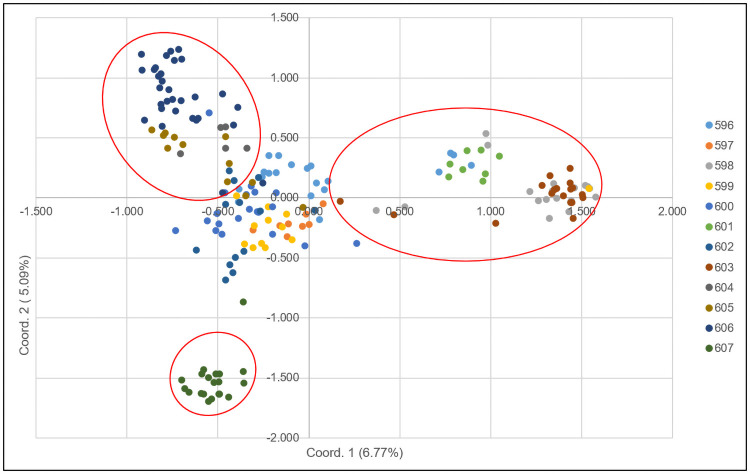
The principal coordinate analysis (PCoA) using genetic distance.

A total of eight genetic clusters were discerned from UPGMA clustering for the 186 Nigerian palms (Fig 1). Mixture of palms from various populations were segregated out to form cluster I (596/14, 596/32, 599/9, 600/36, 605/9, 605/11, 605/16, 606/4 and 606/56). Clusters II and III consisted of majority of palms from pop. 599 and 597, respectively. Palms from pop. 604, 605 and 606 were segregated to form cluster IV while palms from pop. 598, 601 and 603 grouped as cluster V. Cluster VI was formed by palms from pop. 596. Population 600 was in cluster VII and pop. 602 and 607 grouped as cluster VIII. When PCoA was applied to study genetic relatedness, the first two coordinates accounted for only 11.86% of total genetic variation observed and was not strong enough to stratify palm segregation (Fig 2). Nevertheless, the PCoA plot clearly revealed segregation of pop. 598, 601 and 603 as a cluster whereas pop. 604, 605 and 606 were grouped as another cluster. Distinct demarcation of pop. 607 from the other populations was also observed from this analysis.

**Fig 3:**
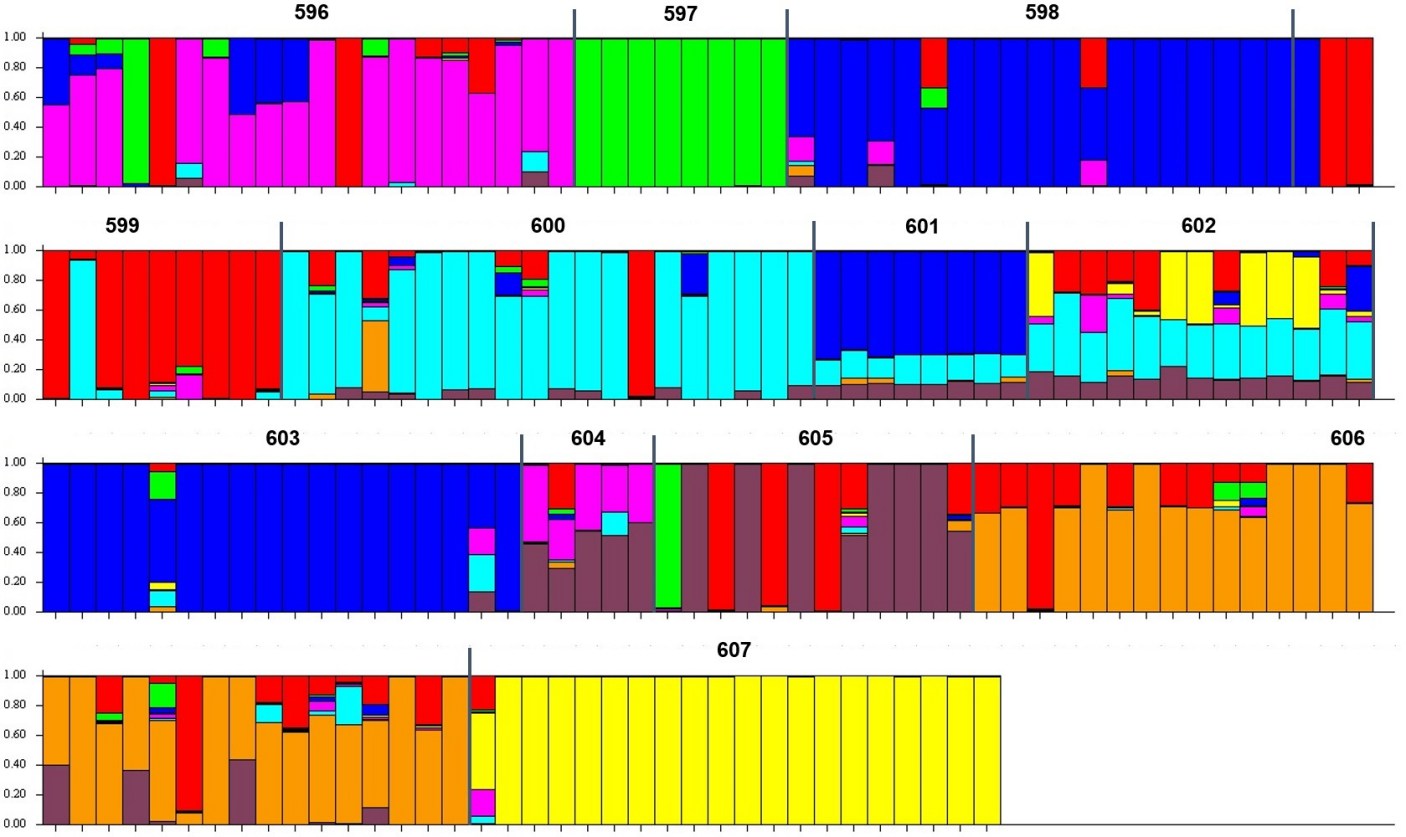
Bayesian model-based analysis using STRUCTURE software (*K* = 8).

The result of Bayesian clustering analysis largely agreed with the distance-based cluster analysis. The estimate of likelihood of data, Δ*K*, reached its maximum value when *K* = 8, classifying the Nigerian-based populations into eight genetic groups (Fig 3). Population 596 (pink), 597 (green), 600 (turquoise), 606 (orange) and 607 (yellow) formed five distinct clusters. Populations 598, 601, 603 grouped as a separate cluster (blue) and another cluster formed by some palms from pop. 605 (maroon). Population 599 and several palms of various populations grouped as the red cluster whereas pop. 602 and 604 were revealed to be highly admixed. The population structure result was generally well supported by UPGMA cluster analysis. Therefore, this study truly revealed the genetic relationship of the Nigerian-based populations.

### Development of core set

Both PowerCore and Core Hunter established core collections retained all the 928 alleles from the Nigerian-based germplasm with 65 (34.9% of the total population) and 58 (31.2%) palm identified, respectively (Tables 5 and 6). Various sampling intensity was tested in Core Hunter (20–35%) with perfect allelic coverage (CV = 1) attained when sampling size was 31%. Both software had captured different palms as the core set with 45 common palms detected (Table 5). The highest number of core palms in both core sets were from pop.606, so did the highest number of common core palms. This is most likely attributed to the highest number of private alleles in pop. 606 (*PA* = 33). Core Hunter outperformed PowerCore with lesser number of palms shortlisted. Expected heterozygosity (*H*_*e*_) and average genetic distance between entries (E-E) were comparable for both core collections (Table 6). PowerCore produced more representative core set compared with Core Hunter because its A-NE value was smaller (PowerCore = 0.217; Core Hunter = 0.242). In contrast, Core Hunter scores better than PowerCore in terms of diversity (higher average and minimum E-NE). The minimum E-NE value was significantly lower in the core set assembled by PowerCore (0.190) compared with Core Hunter collection (0.344).

**Table 5:** Summary of core collections developed using PowerCore and Core Hunter.

Population	Number of palms	Number of core palms selected by PowerCore	Number of core palms selected by Core Hunter	No. of common palms between both core sets
596	20	7 (35)	7 (35)	3 (15)
597	8	4 (50)	4 (50)	2 (25)
598	19	1 (5.3)	2 (10.5)	0 (0)
599	12	7 (58.3)	5 (41.7)	5 (41.7)
600	20	7 (35)	7 (35)	7 (35)
601	8	1 (12.5)	1 (12.5)	1 (12.5)
602	13	7 (53.8)	7 (53.8)	6 (46.2)
603	18	3 (16.7)	2 (11.1)	1 (5.6)
604	5	2 (40)	2 (40)	2 (40)
605	12	5 (41.7)	4 (33.3)	2 (16.7)
606	31	17 (54.8)	14 (45.2)	13 (41.9)
607	20	4 (20)	3 (15)	3 (15)
**Total**	186	65 (34.9)	58 (31.2)	45 (24.2)

The values in parentheses are percentage of palms per population.

**Table 6:** Comparison of core collections developed using PowerCore and Core Hunter based on Bray-Curtis genetic distance measures.

Core Collection	N	CV	H_e_	Mean A-NE	Mean E-E	Min E-NE	Mean E-NE
PowerCore	65	1	0.539	0.217	0.633	0.190	0.398
Core Hunter	58	1	0.542	0.242	0.632	0.344	0.434

*N* = Number of palms; *CV* = Allele coverage; *H*_*e*_ = Expected heterozygosity; *A-NE* = Genetic distance between each accession in the original collection and the nearest entry in the core set; *E-E* = Genetic distance between entries; *E-NE* = Genetic distance between each entry and the nearest neighbouring entry.

**Fig 4:**
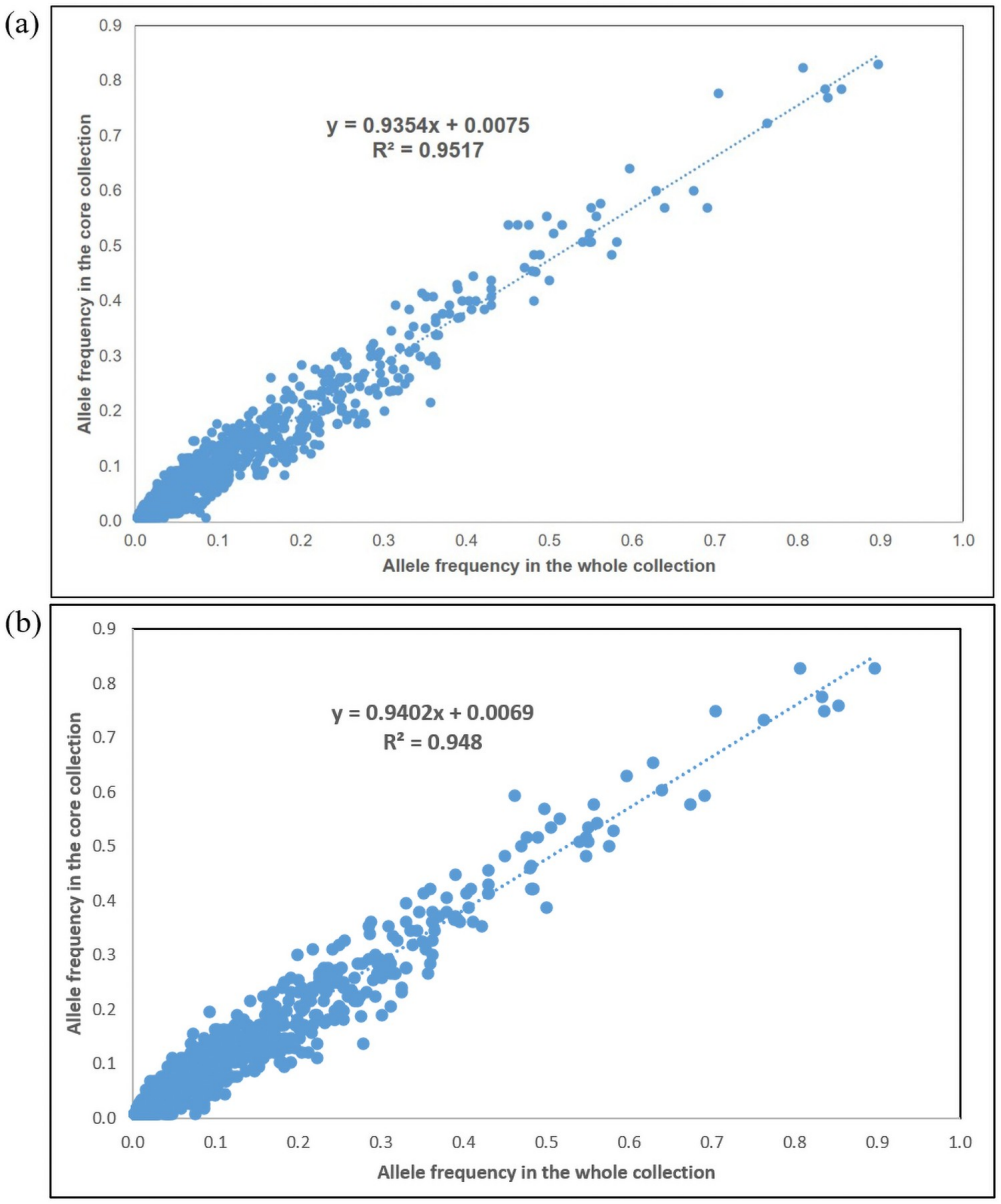
Correlation between allele frequency of the 928 SSR alleles in the core sets and in the whole collection (186 palms). (a) Core set developed using PowerCore (65 palms); (b) Core set developed using Core Hunter (58 palms).

**Fig 5:**
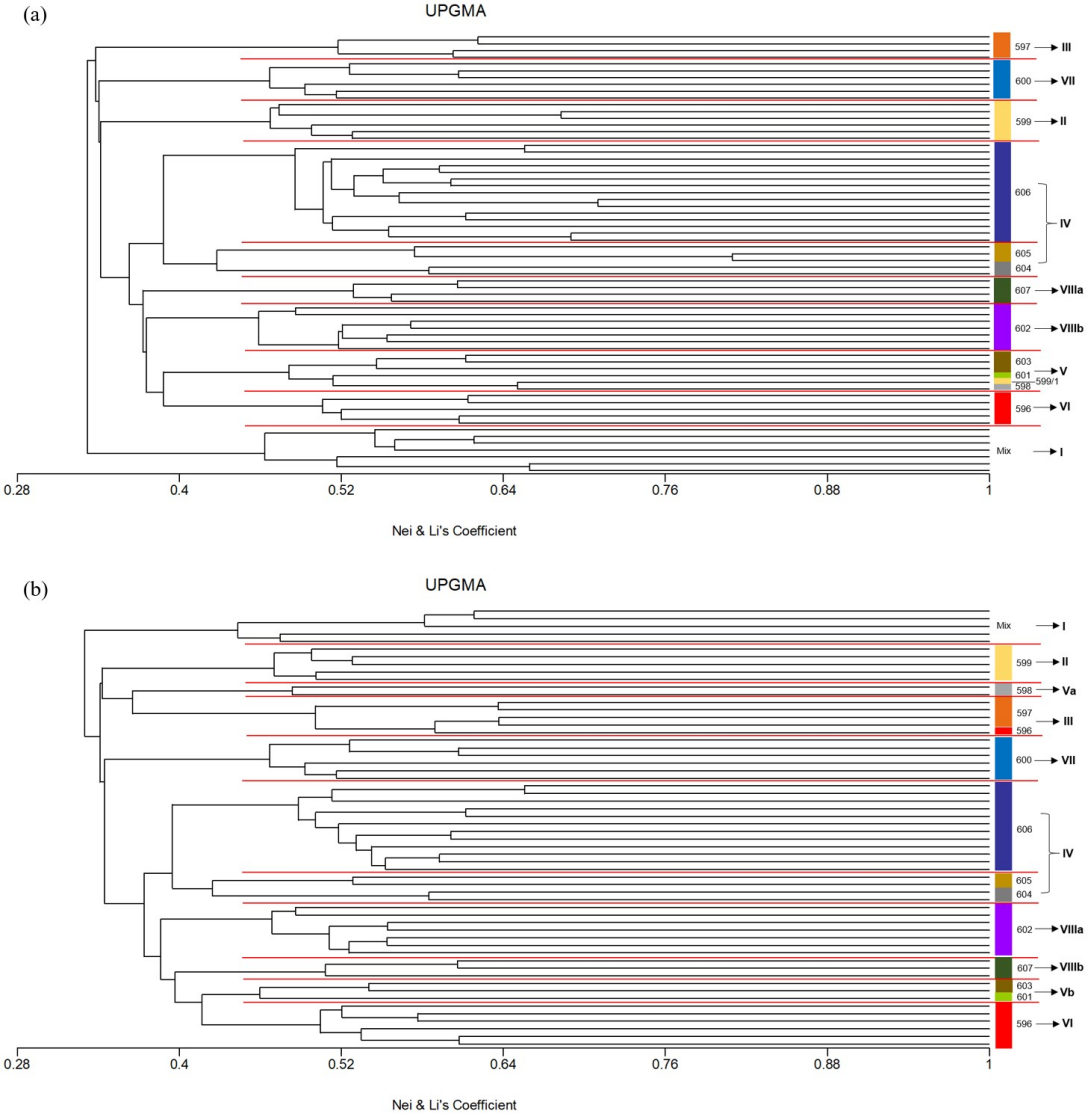
UPGMA clustering of the core collections based on Nei and Li’s coefficient. (a) Core collection developed using PowerCore; (b) Core collection developed using Core Hunter.

In addition, t-test (p<0.05) showed that there was no significant difference in the genetic diversity indexes calculated between the germplasm genotyped and the identified core collections using PowerCore and Core Hunter (Table 7), suggesting maximum allelic diversity was preserved in the reduced population size of both core collections. Both core sets also performed equally well in genetic diversity with no significant difference in the genetic diversity parameters. Meanwhile, no significant differences in allele frequency for all 107 loci as evaluated by chi-square test (p<0.05) and a high correlation of allele frequencies were observed between both core sets and the entire germplasm (R^2^ = 0.952 and 0.948 for core sets obtained by PowerCore and Core Hunter, respectively) (Fig 4). UPGMA clustering of the two core sets revealed highly similar genetic structure as the whole collection (Fig 5). A total of nine and ten different genetic groups were discerned for core sets assembled by PowerCore and Core Hunter, respectively. Slight discrepancy was observed where core palms from pop. 602 and 607 were segregated into two closely-related groups for both core sets whereas pop. 598 was also separated into one cluster in core set assembled by Core Hunter. Nevertheless, the genetic structure of the Nigerian germplasm was generally well maintained by both the core collections and further confirming their representativeness. Based on all the evaluation criteria, Core Hunter captured the best core set with smaller population size, selected entries of good representativeness, genetically more distant as well as maintaining the genetic diversity and structure of the original Nigerian germplasm.

**Table 7:** Comparison of genetic diversity indexes between core collections developed using PowerCore and Core Hunter against the entire Nigerian-based germplasm.

	N	TA	A	A_e_	I	H_o_	H_e_	F
**Total populations**	186	928	4.290 (0.050)	2.777 (0.030)	1.082 (0.012)	0.644 (0.008)	0.576 (0.005)	-0.110 (0.009)
**Core collection by PowerCore**	65	928	3.520 (0.048)	2.609 (0.031)	0.973 (0.013)	0.659 (0.009)	0.539 (0.006)	-0.238 (0.013)
**Core collection by Core Hunter**	58	928	3.461 (0.046)	2.598 (0.030)	0.973 (0.012)	0.655 (0.009)	0.542 (0.006)	-0.220 (0.012)

*N* = Number of palms; *TA* = Total number of alleles; *A* = Number of different alleles; *A*_*e*_ = Number of effective alleles; *I* = Shannon’s Information Index; *H*_*o*_ = Observed heterozygosity; *H*_*e*_ = Expected heterozygosity; *F* = Fixation index. The values in parentheses are standard errors.

## Discussion

Plant breeding still relies, mainly, on the exploitation of existing natural variation to broaden the genetic base of current commercial planting materials. Oil palm is being considered as orthodox seed and not recalcitrant, but the seeds can only be stored for 2 years with gradual reduction in germination rate [42]. Oil palm germplasm are generally maintained as field genebanks with their genetic variation being evaluated via morphological and agronomical traits analysis. Molecular genetic tools would allow assessment of genetic diversity of the germplasm and facilitate development of effective crop improvement strategies.

An average of 8.67 alleles per microsatellite locus was scored in the present study with an effective number of marker alleles per locus of 2.78. This is lesser than the average number of alleles (14.5 and 13.1 alleles/locus, respectively) revealed by molecular characterization of oil palm germplasm collected from Africa and Latin America [14, 43]. The difference in alleles number could be due to variation in the origin of sample from different germplasm collections. Wild germplasms collected from open-pollinated bunches have pronounced genetic variability contributing to higher allele number per locus compared to descendent materials generated from sib-mating or selfing in our germplasm collection. In addition, the difference in allele numbers may also arise from the smaller sample size, different number and/or different microsatellite markers used in our work.

Extensive molecular studies have been conducted on the MPOB oil palm germplasm with comparatively high variation noticed in Nigerian collection, indicating that it is a possible centre of diversity for *Elaeis guineensis* oil palm [8, 10, 11]. High heterozygosity value was obtained in the present study (*H*_*e*_ = 0.576), which was higher than those reported for MPOB Nigerian germplasm based on isozymes (*H*_*e*_ = 0.15–0.244 with *H*_*e*_ of population 12 = 0.187 by [10]), RFLP (*H*_*e*_ = 0.228 by [11]) and EST-SSR (*H*_*e*_ = 0.442 by [44]; *H*_*e*_ = 0.534 by [18]), but lower than the one published by [14] using SSR (*H*_*e*_ = 0.712–0.803 with *H*_*e*_ of population 12 = 0.736). The number of effective alleles calculated from our collection (*A*_*e*_ = 1.964–3.343, average = 2.777) was also lower than those reported by [14] (*A*_*e*_ = 2.9–3.6 with *A*_*e*_ of population 12 = 3.2). Despite being descendants of MPOB Nigerian population 12, high genetic variation and prevalence of private alleles in all population in the current AAR’s Nigerian-based germplasm indicate that they are invaluable resources for introgression into commercial planting material. These results also suggest that the set of SSR used is very polymorphic and robust for diversity studies of oil palm.

Fixation index, *F*, is a measure of the excess or reduction in heterozygosity of an individual due to non-random mating within the germplasm [45]. Despite having small population size and mating nature of selfing or cross between relatives, majority of the populations assayed has negative *F* suggesting high level of out-crossing prevailed. Only pop. 605 exhibits excessive homozygosity (*F* = 0.161), indicating inbreeding due to self-mating nature. Negative *F* values were also observed in MPOB Nigerian germplasm [10, 44], again indicating the high genetic variation in both the original Nigerian germplasm and current descendant collection. Both moderate and high genetic differentiation among populations (pairwise population *F*_*ST*_ ranging from 0.066 to 0.202 and gene flow *Nm* = 1.117) as well as close genetic relationship (mean Nei’s genetic distance = 0.466) were revealed in our collection. This was further evidenced by the 71% of variation attributed by genetic differences within population from AMOVA analysis. Significant genetic variations among individual within population was reported in various studies of oil palm, as expected for an allogamous and long-lived perennial species [10, 43, 46]. Diversity study of oil palm’s Angola germplasm using SNP markers revealed as high as 93% of variation came from within-population differences [19] whereas [47] reported an *F*_*ST*_ value of 0.15 among 51 oil palm genotypes from Congo using 7 randomly amplified microsatellite markers RAM indicating substantial genetic difference among populations. Mating among closely-related individuals may have contributed to some degree of gene flow and thus moderate differentiation among population analyzed.

Maintenance of living collections, common for perennial tree crops such as oil palm, is expensive and labour intensive. Establishment of core collections is an efficient approach for germplasm manipulation, reducing the cost while keeping the maximum genetic diversity of the entire germplasm with minimum repetitiveness [48]. Various approaches have been pursued to develop core collections [33, 34, 49–53] and selection of the most suitable evaluation methods depends upon the purpose of core collections [37]. Two different software were adopted in the present study to develop core set, namely, PowerCore software with heuristic search based on advanced maximization (M) strategy [33] and R package Core Hunter with local search algorithm [34] focusing on allelic coverage in this study. Both core collections consist of 65 and 58 palms with representation from all 12 populations, encompassing 34.9% and 31.2% of the entire collection, respectively. These core sets have lesser number of palms than the one reported for oil palm accessions from Angola, Cameroon and Deli Dura from Indonesia (289 core entries from 788 accessions studied (37%) with total genetic diversity *H*_*T*_ = 0.759) [13] but larger than the core set for *E*. *oleifera* collection with origins from South and Central America (34 over 532 palms assayed (6.4%) with overall mean *H*_*e*_ = 0.221) [16]. The final size of core collection largely depends on the levels of variability and redundancy in the collection, resources available for maintenance of the core set and regeneration frequency of the species and therefore, no single size will be appropriate for all cases [54, 55]. In this study, Core Hunter successfully captured a core set with 7 accessions fewer than PowerCore with both having prefect allelic coverage. The superiority of Core Hunter in developing significantly smaller core sets has previously been reported [36].

Genetic distance-based criteria were proposed to evaluate core collections as they are intuitive to understand and both representativeness of accessions and diversity within core collections were explored [37]. Criterion A-NE is minimized to produce core sets that maximally represent all individual accessions from the whole collections. Value of A-NE decreases when the size of core sets increases as entries of larger core sets fill the gap of the smaller core sets decreasing the average distance between accessions of the original collection and the selected entries in the core sets [56]. Thus, PowerCore found core set with smaller mean A-NE than Core Hunter. Meanwhile, maximizing E-E allows construction of highly diverse core sets but might lead to over-representation of extreme values at one end of the core sets which could be further distinguished using minimum and mean E-NE criteria [37, 57]. High mean E-E and low E-NE distance for collection assembled by PowerCore indicate the presence of genetically similar entries at diverse end of the core set. Core Hunter performed better for these criteria. The default sampling intensity of Core Hunter is 20% and this criterion was relaxed in the present study in order to select core set that preserve all alleles present in the Nigerian germplasm. This was achieved when sampling intensity reached 31%. Core Hunter outperformed PowerCore then when it found a core set that was simultaneously representative, high genetic distance and diversity, high heterozygosity and most importantly smaller core size for more efficient germplasm conservation.

Additionally, both core sets obtained were also validated and concurred with the selection criteria presented in other studies [58–60]. Firstly, the core sets retain all the 928 alleles present in the original collection and guarantee the preservation of rare alleles, which is critical for maintaining the genetic diversity of the collection. Rare alleles are important for plant breeding as they may have unique genetic potential or adaptive values [61]. Second, there are no significant differences in genetic parameters (*A*, *A*_*e*_, *I*, *H*_*o*_, *H*_*e*_, *F*) between both core collections and the original Nigerian populations with increased negative value of *F*, albeit insignificant. This was expected because the diversity increases with elimination of genetically similar accessions during core set development [62]. Third, the allele distribution of all loci in the core sets is highly comparable and correlated with the whole collection, indicating that they contain not only the same alleles but also represent nearly identical allele frequencies. Lastly, validation based on cluster analysis suggests the core sets generally maintain and represent the genetic structure of the entire population [63]. Collectively, the selected core set from Core Hunter is the best and thus valuable for conservation purpose.

Field genebank allows an easy and ready access for utilization. However, living collection is not the best route for long term conservation as potential loss may occur due to extreme weather conditions and pest and diseases such as basal stem rot in oil palm caused by the fungal pathogen *Ganoderma boninense* [64, 65]. Besides, the aging germplasm may become inaccessible for crossing. For instance, the Nigerian-based populations under study were planted more than 25 years and as of to-date, excessive palm height has had hindered pollen collection and controlled pollination of some of the populations in the collection. Currently, there is no official publication on the optimal strategy to save and conserve the collected oil palm germplasm to the next-generation. The conventional approach is to pool pollen collected from all the palms to conduct controlled pollination on a few selected palms in the collection. The disadvantages of this approach are loss of pedigree information of the progenies and potential loss of genetic diversity. The genetic variation of the germplasm may not be captured fully and the risk of having redundancy is high. Assessment using molecular markers to estimate genetic diversity and to gain understanding on population structure will be of great help to formulate strategy to conserve maximal diversity with minimal redundancy. This serves the purpose of identifying the core set via molecular approach in this study.

The focus of germplasm conservation for oil palm can be shifted from whole collection to the identified core set. One of the possible ways to conserve germplasm is to clone the identified core set via tissue culture. This allows precise conservation of maximum genetic diversity with minimum redundancy and zero genetic erosion. The number of ramets to be planted in the field can be lower, more manageable and cost-saving than progeny trials. However, oil palm tissue culture is not widely implemented, making up only 2% of commercial planting material, due to its long and labour intensive process, low efficiency of culture amenability and high risk of somaclonal variation [66, 67]. Depending on the genotype used, the rate of callogenesis and embryogenesis of oil palm tissue culture explants ranged between 12–27% (average 17%) and 0–36% (average 4%), respectively [68]. Responses also depend on the age of ortet palms and the type of explant used, in which adaptation of culturing protocol might be required but deviation from established protocols is unfavorable for the fear of increasing risk of somaclonal variation [67]. Leaf explants from old palms, such as the 25 years old germplasm under study, is likely unresponsive to tissue culture treatment. Another difficulty of clonal propagation lies in achieving true-to-type reproduction of ortet palms, especially with the incidence of mantled abnormality. Although mantling level in the field has been generally maintained at tolerable level of less than 5% due to stringent process quality control and planting of a package of different clones rather than single clone planting in the field, the frequency and degree of abnormalities are highly variable between genotype, palms of the same clone and flowers of the same palm as well as *in vitro* culture conditions [68, 69]. Mantled fruit abnormality, normally parthenocarpic, can only be visually detected when the ramets produce the first few inflorescences at 1.5 to 2 years after field planting, leading to waste of resources (planting area, labour and fertilizer). Immature leaf explants, unmerged leaves grouped above single apical meristem of oil palm, are typically being used for production of tissue culture plantlets. The ortet is injured during de-spearing and it takes 3–5 years for the palms to recover. In worst case, the apex is damaged during sampling, leading to total loss of valuable palms [67]. The chances of old palms to survive the aggressive de-spearing process are still in doubt. With the various difficulties associated with clonal propagation of oil palm, it remains daunting to regenerate the core set via tissue culture for conservation purpose.

A more practical breeding approach is to re-create the germplasm through selfing or intercrossing of the core set that was identified through molecular analysis. With known genetic diversity and relationship of the core set, this approach stands a better chance to capture the diversity of the original germplasm collection. However, there is little disadvantage in term of land size and expenditure required to maintain the living collection in the field. The intercrossing of 58 core palms into 29 populations within each genetic cluster via punnet square design for the present study would require 13.4 ha of land for planting. [16] suggested 20–30 seeds may be sufficient to preserve the genetic diversity in the original population. By using the genetic diversity of the core set as reference, the selection of the 20–30 seedlings could be guided by their molecular profiles. This will ensure maximum diversity is being conserved in the next generation.

The use of molecular markers for the development of core collection is advantageous as they can accurately reflect the genetic diversity regardless of plant growth status, developmental and environment effects [70]. Population persistence and resilience to environmental changes are usually positively correlated with genetic diversity [71]. [16] proposed to combine information from both molecular marker and phenotypic data for selection of core collections in oil palm germplasm to ensure traits of interest are incorporated into the next generation for future access while retaining the overall genetic variability for selection gains in the future. A composite core collection has been reported for oilseed crop, Safflower, that includes data on molecular variability as well as phenotypic parameters to avoid trade-off between diversity captured using both data sets [72]. Nevertheless, the current core set for oil palm based on genetic information only could be further evaluated using morphological data to assess the retention of phenotypic variability [37, 73].

## Conclusion

The present study reported SSR marker-based molecular assessment on genetic diversity and population structure of a Nigerian-based oil palm field genebank in AAR. High levels of genetic heterozygosity were detected despite being descendants of MPOB Nigerian germplasm population 12, indicating this material is invaluable for introgression into current breeding programme. Two different software were compared for their effectiveness to develop core collection of Nigerian population and Core Hunter version 3.2.1 outperformed PowerCore 1.0. Core Hunter constructed a core collection of 58 palms, accounting for 31.2% of the total population. This is the first report describing the molecular characterization and validation of core set establishment for oil palm germplasm material. The core set captures all the alleles with genetically distinct entries while maintaining intact genetic diversity and structure of the original population. This core collection would be of great interest to breeders for exploitation of the prevalent diversity in crop improvement programs as well as effective conservation of the field genebank: a step towards molecular-assisted germplasm conservation.

## Supporting information

S1 Table Summary information of the 107 Simple Sequence Repeat (SSR) markers used to genotype the 186 Nigerian-based germplasm collection. (XLSX)

S2 Table Genetic diversity calculated for the 107 SSR markers. (XLSX)
